# Study of myoblast differentiation using multi-dimensional scaffolds consisting of nano and micropatterns

**DOI:** 10.1186/s40824-016-0087-x

**Published:** 2017-01-11

**Authors:** Sung Ho Cha, Hyun Jong Lee, Won-Gun Koh

**Affiliations:** Department of Chemical and Biomolecular Engineering, Yonsei University, 50 Yonsei-ro, Seodaemun-gu, Seoul, 120-749 South Korea

**Keywords:** Myogenesis, Multi-dimensional scaffold, Topographical cue, Electrospun alignment, Poly(ethylene glycol) hydrogel pattern

## Abstract

**Background:**

The topographical cue is major influence on skeletal muscle cell culture because the structure is highly organized and consists of long parallel bundles of multinucleated myotubes that are formed by differentiation and fusion of myoblast satellite cells. In this technical report, we fabricated a multiscale scaffold using electrospinning and poly (ethylene glycol) (PEG) hydrogel micropatterns to monitor the cell behaviors on nano- and micro-alignment combined scaffolds with different combinations of angles.

**Results:**

We fabricated multiscale scaffolds that provide biocompatible and extracellular matrix (ECM)-mimetic environments via electrospun nanofiber and PEG hydrogel micro patterning. MTT assays demonstrated an almost four-fold increase in the proliferation rate during the 7 days of cell culture for all of the experimental groups. Cell orientation and elongation were measured to confirm the myogenic potential. On the aligned fibrous scaffolds, more than 90% of the cells were dispersed ± 20° of the fiber orientation. To determine cell elongation, we monitored nuclei aspect ratios. On a random nanofiber, the cells demonstrated an aspect ratio of 1.33, but on perpendicular and parallel nanofibers, the aspect ratio was greater than 2. Myosin heavy chain (MHC) expression was significantly higher i) on parallel compared to random fibers, ii) the 100 μm compared to the 200 μm line pattern. We confirmed the disparate trends of myotube formation that can be provoked through multi-dimensional scaffolds.

**Conclusion:**

We studied more favorable environments that induce cell alignment and elongation for myogenesis by combining nano- and micro-scale patterns. The fabricated system can serve as a novel multi-dimensional platform to study in vitro cell behaviors.

## Background

Skeletal muscle is a highly organized structure with long parallel bundles of multinucleated myotubes that are formed by the differentiation and fusion of myoblast satellite cells [1]. It has also been previously shown that the aligned structures allow myoblasts to form myotubes [2]. Therefore, to achieve successful regulation and differentiation of skeletal muscular cells in vitro*,* well designed and physiologically aligned architectures need to be developed, which remain an ongoing challenge.

There have been numerous attempts to provide suitable topographical cues in cell culture systems for cellular orientation control and the enhancement of cell-cell interactions for better myotube formation [2–4]. In particular, the topographical scale is hinged on the fabrication method, such as at the nano- and micro-scale, and it is a key parameter to better understand cellular behaviors.

Micro-scale topography has been generated via reactive ion etching of a substrate [5], pattern transfer in soft lithography [6–9] and PEG hydrogel patterning [9, 10]. Above all, the PEG hydrogel patterning technique, which was used in this study, has the advantages of convenience, cost effectiveness, and an easy procedure [9, 10]. For nano-scale topographic surfaces, the electrospinning technique, which provides conditions that resemble the physical structure of native collagen fibrils or extracellular matrix (ECM) [11, 12], has been widely used. Although we can obtain random fibrous structures through conventional electrospinning, the nanofiber aligned structure can be fabricated by winding fibers over a rotating cylinder.

In previous studies, nano- or micro-scale pattern techniques and structures were applied to cell scaffolds independently [13–18]; therefore, the composite effects of the multiscale have not been observed. In this report, we fabricated a dual-scaled cell culture system using electrospinning and PEG hydrogel micropatterning with different combinations of angles. Myoblasts were cultured on the fabricated scaffolds, and skeletal muscular changes were observed with regard to the fiber alignment and angles between the nano- and micro-axes.

## Methods

### Materials

Poly(ethylene glycol) diacrylate (PEG-DA, MW 575), 2-hydroxy-2-methylpropiophenone (HOMPP), polycaprolactone (PCL, MW 80000), dimethylsulfoxide (DMSO), ethanol, Dulbecco’s Modified Eagle’s Medium (DMEM), fetal bovine serum (FBS), horse serum (HS) were purchased from Invitrogen (Carlsbad, CA, USA). 3-(4,5-dimethylthiazol-2-yl)-2,5,diphenyltetrazolium bromide (MTT) antibiotic/antimycotic solution, and trypsin/ethylenediaminetetra-acetate (trypsin/EDTA) were purchased from Sigma-Aldrich (Milwaukee, WI, USA). C2C12 mouse myoblasts were purchased from the Korean Cell Line Bank (Seoul, Korea). Phosphate buffered saline (PBS, 0.1 M, pH 7.4) was purchased from Invitrogen (Carlsbad, CA, USA). Mouse monoclonal antibody to MHC (sc-376157) and goat anti-mouse IgG-FITC (sc-2010) were purchased from Santa Cruz (Dallas, TX, USA). Photomasks for photolithography were prepared using AutoCAD and were printed on transparencies using a standard laser jet printer (LaserWriter 16/600 PS, Apple, Inc., Cupertino, CA, USA).

### Scaffold fabrication

The electrospinning apparatus used in this study consisted of a plastic syringe (10 mL) capped in a flat-ended 23 G metal needle, a syringe pump (KD Scientific, Holliston, MA, USA) for controlling the feeding rate, a stainless steel substrate as a collecting plate, and a high voltage power supply (NanoNC, Seoul, Korea). PCL polymer solutions (20 wt%) were prepared by diluting the polymer solutes in trifluoroethanol. The solution was transferred to a syringe for electrospinning. To obtain electrospun PCL fibers, a 7.0 kV positive voltage was applied to the solution via the needle, and a constant feeding rate of solution (0.5 mL/h) was provided by the syringe pump. The distance between the tip of the needle and collecting plate was 15 cm. The electrospun fibers were collected on clean aluminum foil (connected to the ground). The resultant PCL fibers were then treated with oxygen plasma (Femto Science, Kyunggi, Korea) for 10 min prior to the hydrogel patterning process. The radiofrequency power and pressure of the plasma treatment were 40 W and 0.1 mmHg, respectively.

The resultant electrospun fibers were micropatterned with PEG hydrogel using photolithography as described in previous studies [19]. The gel precursor solution was prepared by adding 100 μL of HOMPP as a photoinitiator to 5 mL of PEG-DA in water. This precursor solution was dropped onto the electrospun fibers and spread into a thin layer by covering with a photomask. The precursor solution was then exposed to 365 nm and 300 mW/cm^2^ ultraviolet (UV) light (EFOS Ultracure 100 ss Plus, UV spot lamp, Mississauga, Ontario, Canada) for 1 s through the photomask. The morphology of the micropatterned fibrous scaffold was observed by scanning electron microscopy (SEM) (Hitachi Model S-4200 at 30 kV, Nissei Sangyo Co., Tokyo, Japan). For the cell studies, the scaffolds were sterilized in a 70% v/v ethanol solution for 30 min and then washed five times with PBS to remove any traces of ethanol.

### Cell culture and seeding

C2C12 myoblast cells were cultured in DMEM containing 4.5 g/L glucose, 10% FBS, and a 1% antibiotic/antimycotic solution. The cells were then incubated at 37 °C in 5% CO_2_ and 95% air. To seed the cells onto micropatterned PCL fibers, both cells were trypsinized from routine culture and centrifuged at 1200 rpm and 25 °C for 5 min. The supernatant was removed, and the cells were re-suspended in fresh culture medium containing serum. An aliquot was obtained for cell counting in a hemocytometer to adjust the seeding density. Finally, approximately 3.0 × 10^4^ cells were seeded onto the micropatterned fibrous scaffolds for proliferation studies. After 5 h, cells containing micropatterned nanofibers were transferred to new 24-well plates to exclude the effect of cells that adhered to the well plate. After 48 h in growth medium, myoblast cultures reached confluence, and the cultures were then switched to differentiation medium for the differentiation study. The differentiation medium consisted of DMEM in addition to 2% horse serum and 1% antibiotic/antimycotic solution.

### MTT assay

MTT assays were performed to investigate the in vitro proliferation of cells cultured on the scaffolds. Briefly, a 10 v/v% MTT solution (5 mg/ml) was added to the culture medium of the cell-seeded scaffolds. The samples were incubated for 1 h at 37.8 °C, and the formazan crystals that were transformed from MTT by mitochondrial reductase were dissolved in DMSO. The absorbance was measured at 540 nm using a microplate reader (Molecular Devices, Sunnyvale, CA, USA).

### Cell orientation and elongation

The cell-laden constructs were observed under an inverted fluorescence microscope (IX53, Olympus Corp., Tokyo, Japan) and analyzed using Image J software. The elongation of C2C12 cells on a nanofibrous scaffold was quantitatively measured by the aspect ratio, which was defined as the ratio between the length of the longest line to the length of the shortest line across the nuclei. The orientation of the cells was determined from DAPI images by measuring the angle between the long axis of the cells and the direction of the scaffolds to generate alignment histograms.

### Immunostaining

For immunostaining, samples were cultured for 2 weeks in differentiation medium. Samples were fixed in 4% paraformaldehyde (Aldrich) in PBS for 15 min and then washed with PBS. The cell membrane was permeabilized with 0.25 v/v% Triton X-100 (Aldrich) in PBS for 10 min. Following permeabilization, the samples were incubated with a mouse monoclonal antibody to the MHC (sc-376157, Abcam) at a 1:100 dilution in PBS overnight at 4 °C. The samples were then incubated in a 1:100 dilution of FITC-conjugated mouse secondary antibody (sc-2010, Abcam) in PBS for 1 h. All of the incubation steps, except the overnight incubation, were performed at room temperature, and the samples were rinsed three times with PBS between each step. The samples were rinsed in PBS and mounted on a coverslip with DAPI (Invitrogen). The samples were cured overnight and imaged with an integrated color CCD camera (Olympus) that was used to obtain fluorescence images.

## Results and discussion

### Fabrication of multi-dimensional scaffolds

**Fig. 1:**
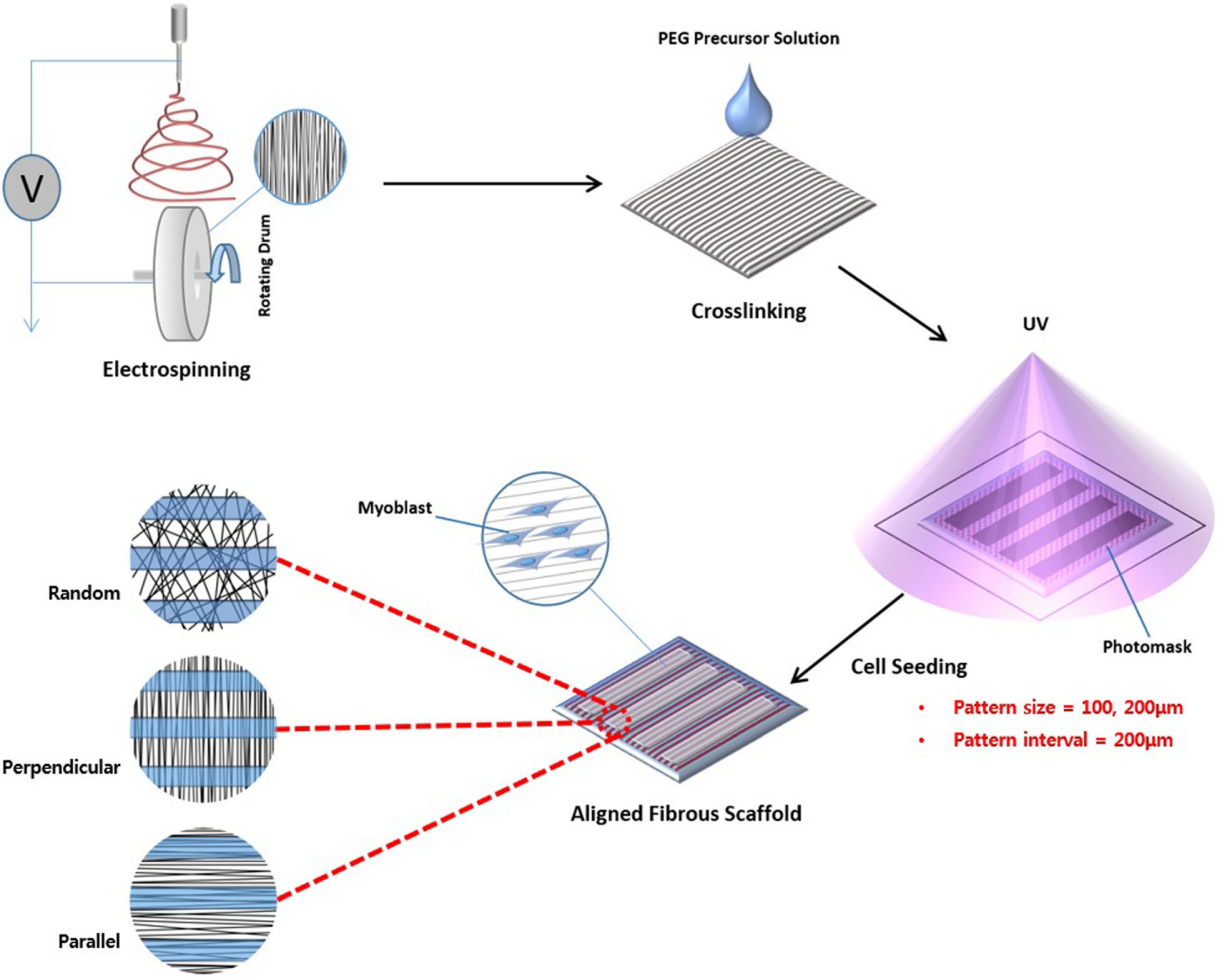
Schematic image of scaffold fabrication

Aligned nanostructures and microstructures were obtained from aligned electrospun fibers and hydrogel patterns, respectively. Figure 1 demonstrates the overall process of scaffold fabrication. Previous research has focused on generating aligned nanostructures or aligned microstructures [20]. However, there have been no other attempts to fabricate a scaffold that has both nano- and micro-scale alignments at the same time. Here, we fabricated a novel scaffold that contained nano- and micro-scale alignments, which allows for the control of the angle between the axis of the two alignments, either in parallel or perpendicular to each other.

**Fig. 2:**
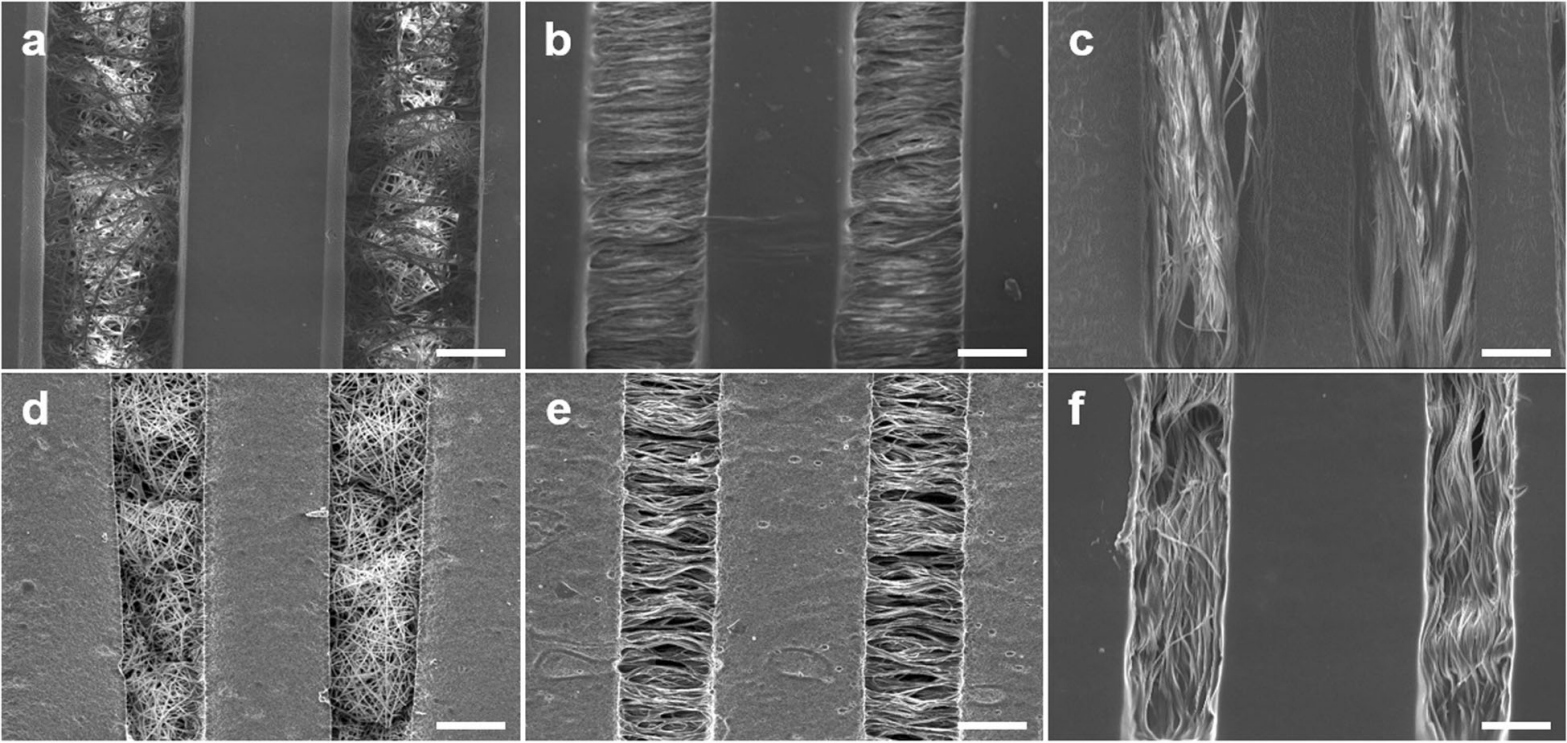
SEM images of fabricated scaffolds (scale bar = 100 μm): 200 μm random (**a**), 200 μm perpendicular aligned (**b**), 200 μm parallel aligned (**c**), 100 μm random (**d**), 100 μm perpendicular aligned (**e**), and 100 μm parallel aligned (**f**)

Random and aligned fibers were applied to observe the effect of the nano-scale topography, and the nanostructures were well incorporated into the PEG hydrogel pattern (Fig. 2). For micro-scale control, the widths of the patterns were set to 200 (Fig. 2a-c) and 100 μm (Fig. 2d-f). There was no damage to the morphologies of the fibers, in particular, the angles between the aligned fibers and micro-line patterns were maintained at 90° (Fig. 2b and e) and 0° (Fig. 2c and f).

### Cell viability and proliferation

**Fig. 3:**
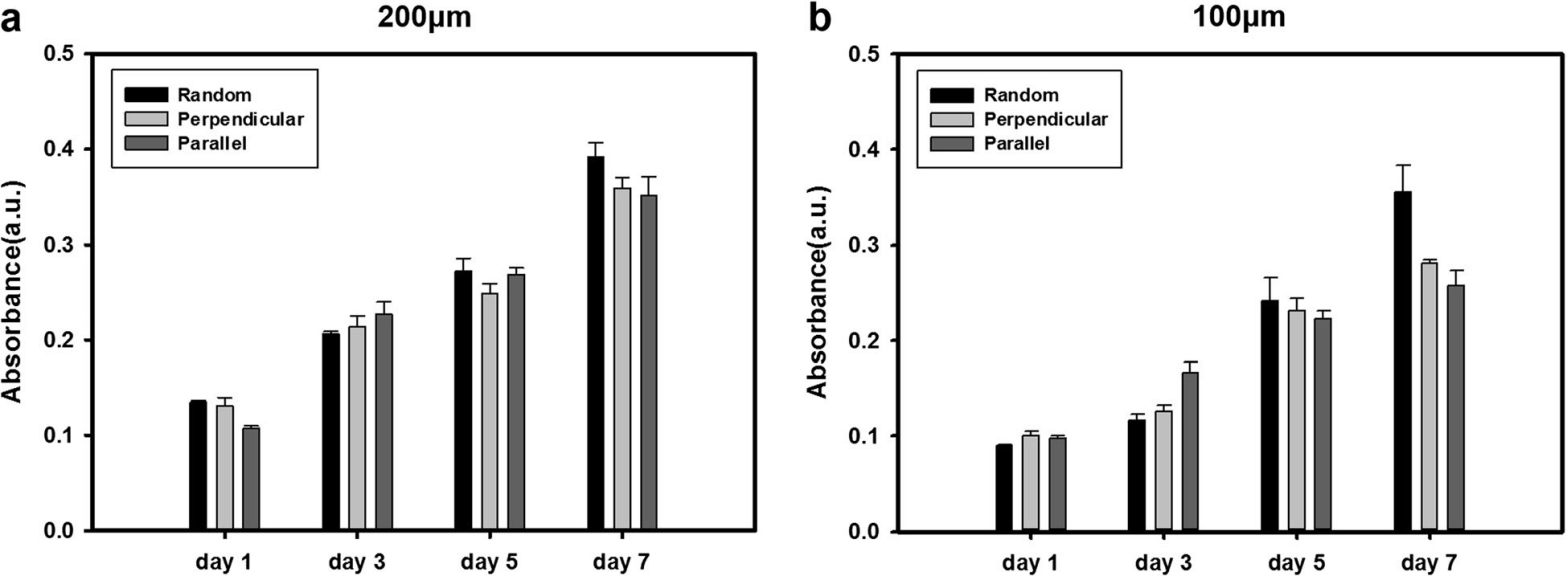
Results of the MTT assay at different periods on 200 μm (**a**) and 100 μm (**b**) patterns

The cell adhesion and proliferation properties of the fabricated scaffolds were confirmed using the MTT assay (Fig. 3). Because PEG hydrogels are known to resist cell adhesion, the resultant micropatterned fibrous scaffolds consisted of two different regions that interact with cells differently: one is the cell adhesion-resistant PEG hydrogel region and the other is the cell adhesion-promoting PCL fiber region. Therefore, cells selectively adhered on the fiber region. According to the day 1 results, cellular adhesion was not significantly influenced by the change in the alignment of the fibers and the width of the hydrogel patterns. Since the absorbance values were gradually increased over 7 days in all cases, the scaffolds were considered to be biocompatible and suitable for myoblast proliferation.

Compared to the pattern size, the MTT assay demonstrated different proliferation trends. Although the absorbance values of the perpendicular and parallel angles were relatively lower than random fibers on day 7, similar levels were generally shown on every day with the 200 μm width (Fig. 3a). However, when cells were cultured on the 100 μm wide pattern (Fig. 3b), cell proliferation increased by a greater extent from day 5 to day 7 for those cultured on random nanofiber scaffolds than cultured on parallel scaffolds. This was probably due to the greater tendency of cells towards differentiation than proliferation that came from parallel topographical cues.

### Cellular alignment and elongation in micropatterned cell-laden hydrogel scaffolds

**Fig. 4:**
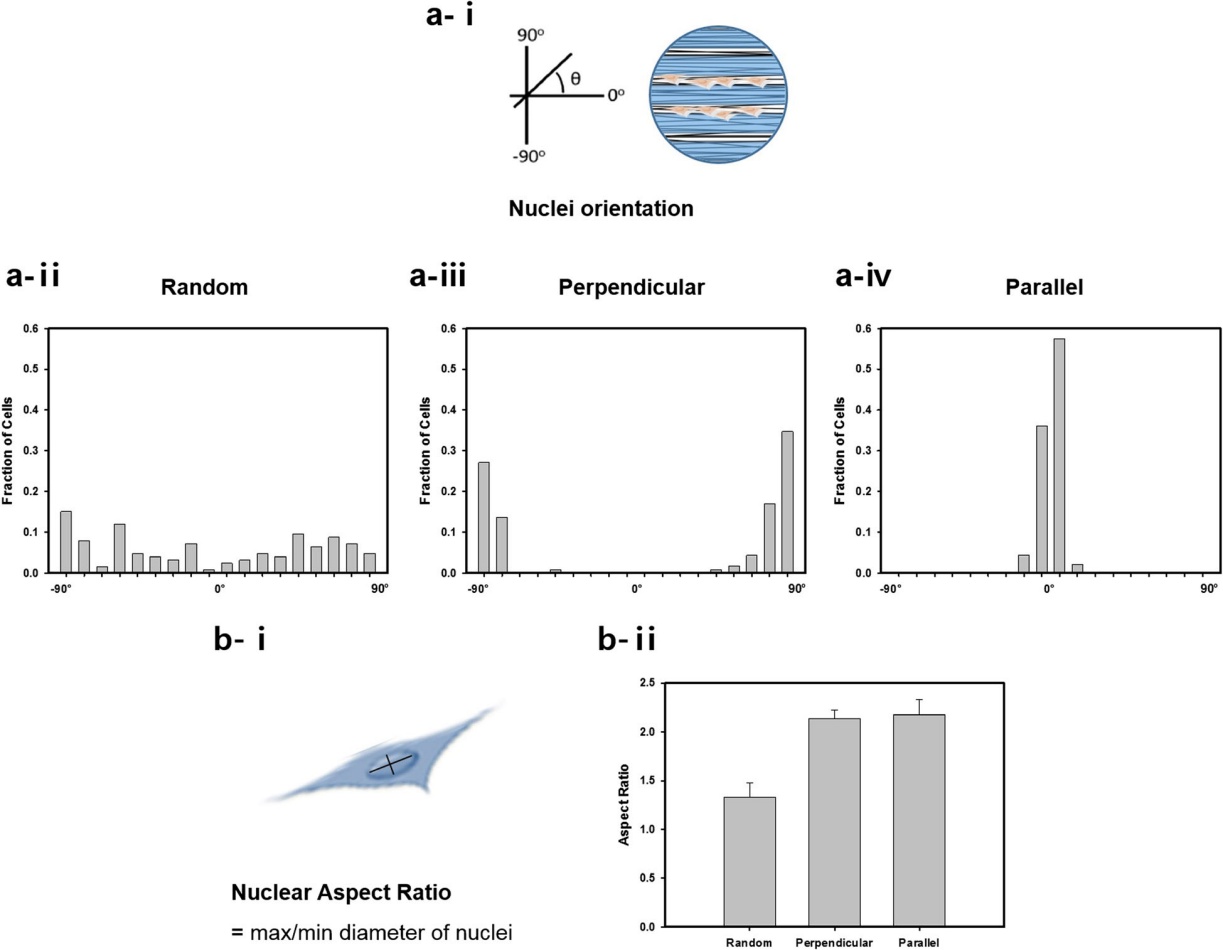
Cell alignment and elongation on scaffolds. **a** Cell alignment was measured by the angle between the long axis of cells and the direction of the micropattern (i); the histograms of the relative alignment in ± 10° increments demonstrate the cellular alignment on the aligned nanofiber (ii-iv). **b** Cell elongation was estimated by the aspect ratio (max/min diameter of nuclei) (i), and the statistical analysis demonstrates that the aspect ratios significantly decreased on the random fibrous scaffolds compared with aligned fibrous scaffolds (ii)

C2C12 myoblasts were cultured and observed on 200 μm fibrous hydrogel scaffolds for 7 days, and modifications in the cell morphology as a result of the surface topography differences were studied (Fig. 4). The orientation of cells cultured on the random nanofiber was randomly spread over the whole scaffold surface. For perpendicularly patterned scaffolds, cells were elongated perpendicularly from the direction of the line pattern, while for parallel patterns, cells were extended along the fibers towards the direction corresponding to the line pattern.

Aligned nanofibers promoted cell alignment along the fiber axis. Because fibers are more compatible with cell adhesion than the hydrogel, the growth of the cells was easily guided by the fiber orientation. Fiber guidance is not the only an impact on cells; hydrogel, which functions as a topographical barrier, also allows cells to stay between the two barriers for a more definite alignment.

To quantitatively represent cellular alignment, we monitored the cell nuclei aspect ratio and orientation after 7 days of cell culture. The nuclei aspect ratio was calculated by dividing the length of the major axis by that of the minor axis (Fig. 4b-i). On random nanofibers, cells demonstrated an aspect ratio of 1.33, but on perpendicular and parallel nanofibers, we observed an aspect ratio greater than 2 (Fig. 4b-ii). From this examination, we affirmed that cells demonstrate greater elongation when aligned in a nanofibrous environment. There was no significant difference between perpendicular and parallel nanofibers (Fig. 4a-iv). From this examination, we affirmed that cells demonstrate greater elongation when aligned in a nanofibrous environment.

Cell alignment was evaluated using Image J software by measuring the angle that each myoblast was elongated with a preferential axis (Fig. 4a-i). The minimum myoblast alignment value of 0° was chosen for unidirectionally oriented myoblasts, while 90° was chosen for perpendicularly oriented myoblasts. Cells that were cultured on random fibrous scaffolds were dispersed over the whole area with arbitrary angles (Fig. 4a-ii). For a case in which the fiber and pattern directions were perpendicularly oriented, more than 70% of the cells were dispersed ± 10° of the fiber orientation (Fig. 4a-iii). For scaffolds with a parallel orientation between the fibers and patterns, more than 90% of the cells demonstrated an angle alignment ± 10° of the fiber orientation. Due to change of nuclei orientations that followed the direction of nanofibers, we confirmed that nano structures influence the cell orientation more than micro structures.

Nanofibers and micropatterns can provide topographical cues for cells and induce cell elongation so that the cells can be neatly aligned along the fiber direction. As a result, not only do the cells express elongated morphology, but an end-to-end configuration also appears; an optimal environment for myogenesis, from myocytes to nascent myotubes, can be provided by the given scaffolds. It was known that the ability to transform between filopodia and small lamellipodia played important roles in directional cell guidance [21]. Filopodia did not show directional extension on patterned substrates prior to spreading, but they transduced topographical cues to the cell to trigger the formation of small lamellipodia along the direction of a microgrooved or parallel nanofiber pattern. The polar lamellipodia formation provided not only a path with directionality, but a driving force for directional cell elongation.

### Myotube formation and cell differentiation

**Fig. 5:**
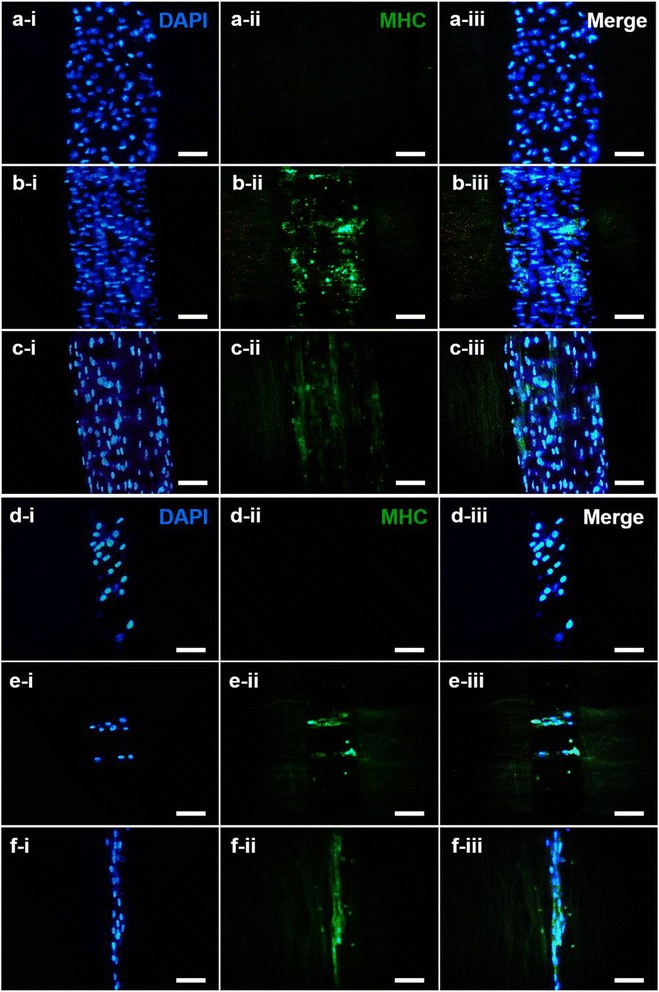
Immunofluorescent images of MHC (*green*) and nuclei (*blue*) for pattern sizes 200 μm (**a**-**c**) and 100 μm (**d**-**f**) (scale bar = 100 μm)

To confirm whether the multiscale scaffold induces differentiation of C2C12 myoblasts, myosin heavy chain (MHC), which is a late-stage differentiation marker of myogenesis, was observed via immunostaining. After inducing differentiation of the cells in differentiation media for two weeks, no MHC expression was observed from myoblasts cultured on a random fibrous scaffold (Fig. 5a-ii, d-ii), but MHC was expressed from those cultured on aligned nanofibrous scaffolds. For the parallel patterned scaffold, nascent myotubes with more than 10 nuclei and a longitudinal length greater than 300 μm within a single perimeter were formed (Fig. 5c-ii, f-ii). However, for the perpendicular pattern scaffold, the overall length was limited to the width of the exposed fiber region, and myotubes, formed by fusion of less than 5 cells, were observed (Fig. 5b-ii, e-ii). In general, on the 200 μm patterned scaffold, MHC expression was observed almost completely over the whole scaffold and more mature myotubes were observed relative to those observed on the 100 μm patterned scaffold.

**Fig. 6:**
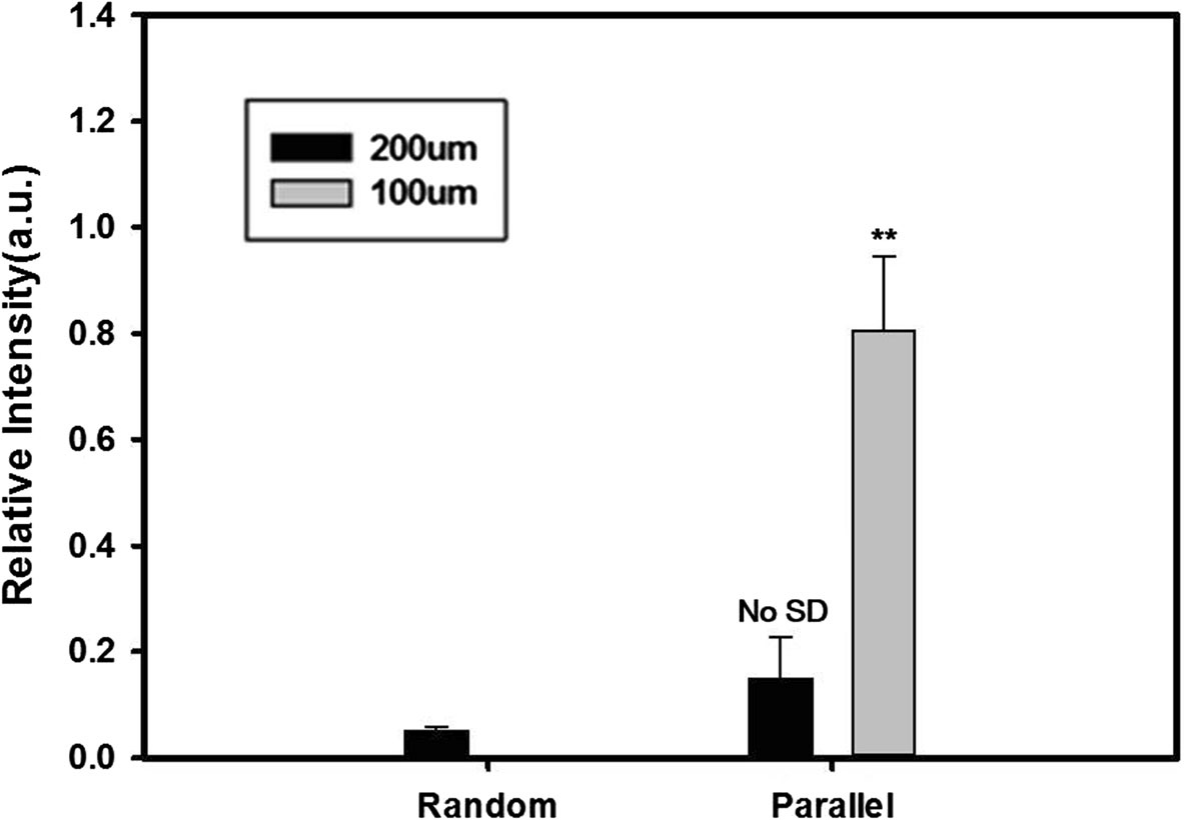
Relative intensity of MHC

To quantitatively analyze the MHC expression, we measured the relative intensity of MHC expression against the cell number, which is represented by the nuclei number (Fig. 6). For both the 200 and 100 μm pattern sizes, more MHC expression was observed on parallel fibrous scaffolds than on the random fibrous scaffolds. Moreover, for random, the difference between 200 and 100 patterns was negligible. However, for aligned, the expression was significantly higher on 100 μm patterns than 200 μm patterns. This indicates that narrower patterns promote myogenesis of myoblasts.

Through this experiment, we demonstrated that multiscale scaffolds provide an environment that can help myoblasts to efficiently differentiate. Moreover, myoblast differentiation is more influenced by nano topography than micro topography; micro topography supplies the nano topography as well as additional topographical cues to cells and can control the length of myotubes, which can indicate the maturity of myotubes.

## Conclusion

In conclusion, we developed nano-micro multiscale matrices to construct implantable scaffolds for the reconstruction of muscle tissue. These scaffolds support the biocompatible environment for cells to survive and provide similar differentiation conditions to native tissue. For a better fusion of myoblasts to myotubes, we used nanofibers and micropatterns to provide topographical cues and aligned physiological architectures. According to the results, cells are more affected by the nano topography of nanofibers; however, with the aid of the structural regulation that comes from micro patterns, the promotion of myotube differentiation could be significantly enhanced. The fabricated system can serve as a novel multi-dimensional platform to study in vitro cell behaviors.

## Abbreviations

DMEM: Dulbecco’s modified eagle’s medium
DMSO: Dimethylsulfoxide
ECM: Extracellular matrix
EDTA: Ethylenediaminetetra-acetate
FBS: Fetal bovine serum
HOMPP: 2-hydroxy-2-methylpropiophenone
MHC: Myosin heavy chain
MTT: 3-(4,5-dimethylthiazol-2-yl)-2,5-diphenyltetrazolium bromide
PBS: Phosphate buffered saline
PCL: Polycaprolactone
PEG: Poly (ethylene glycol)
PEG-DA: Poly (ethylene glycol) diacrylate
SEM: Scanning electron microscopy
UV: Ultraviolet
